# Recent Advances in Obesity-Induced Inflammation and Insulin Resistance

**DOI:** 10.3389/fendo.2013.00093

**Published:** 2013-08-08

**Authors:** Sanshiro Tateya, Francis Kim, Yoshikazu Tamori

**Affiliations:** ^1^Department of Internal Medicine, Division of Diabetes, Metabolism, and Endocrinology, Graduate School of Medicine, Kobe University, Kobe, Japan; ^2^Department of Medicine, University of Washington, Seattle, WA, USA; ^3^Diabetes and Obesity Center of Excellence, University of Washington, Seattle, WA, USA; ^4^Department of Internal Medicine, Diabetes Center, Chibune Hospital, Osaka, Japan

**Keywords:** obesity, chronic inflammation, insulin resistance, adipose tissue, TNFα, macrophages

## Abstract

It has been demonstrated in rodents and humans that chronic inflammation characterized by macrophage infiltration occurs mainly in adipose tissue or liver during obesity, in which activation of immune cells is closely associated with insulin sensitivity. Macrophages can be classified as classically activated (M1) macrophages that support microbicidal activity or alternatively activated (M2) macrophages that support allergic and antiparasitic responses. In the context of insulin action, M2 macrophages sustain insulin sensitivity by secreting IL-4 and IL-10, while M1 macrophages induce insulin resistance through the secretion of proinflammatory cytokines, such as TNFα. Polarization of M1/M2 is controlled by various dynamic functions of other immune cells. It has been demonstrated that, in a lean state, T_H_2 cells, T_reg_ cells, natural killer T cells, or eosinophils contribute to the M2 activation of macrophages by secreting IL-4 or IL-10. In contrast, obesity causes alteration of the constituent immune cells, in which T_H_1 cells, B cells, neutrophils, or mast cells induce M1 activation of macrophages by the elevated secretion of TNFα and IFNγ. Increased secretion of TNFα and free fatty acids from hypertrophied adipocytes also contributes to the M1 activation of macrophages. Since obesity-induced insulin resistance is established by macrophage infiltration and the activation of immune cells inside tissues, identification of the factors that regulate accumulation and the intracellular signaling cascades that define polarization of M1/M2 would be indispensable. Regulation of these factors would lead to the pharmacological inhibition of obesity-induced insulin resistance. In this review, we introduce molecular mechanisms relevant to the pathophysiology and review the most recent studies of clinical applications targeting chronic inflammation.

## Introduction

Obesity develops as a consequence of nutritional excess and insufficient exercise; it causes major adverse health outcomes such as type 2 diabetes, cardiovascular diseases, dyslipidemia, chronic kidney diseases, and cancers, which are serious problems worldwide. These pathological states are strongly associated with insulin resistance or hyperinsulinemia. On the basis of efforts over the last two decades, there have been remarkable developments in the investigation of obesity-induced insulin resistance, especially in terms of the mechanisms involved, some of which are expected to lead to treatments of the disease. Among these, low-grade chronic inflammation in obesity is one of the most innovative and newly identified concepts. The metabolic pathway and the immune response pathway, which are strongly evolutionarily conserved among species, have been found to be strongly associated with each other in the development of obesity-induced insulin resistance. In this review, we look back over the initial findings in the research field of inflammation and insulin resistance and discuss recent studies, including those on clinical applications.

## Obesity-Induced Chronic Inflammation in Adipose Tissue and Adipokine Secretion

Low-grade chronic inflammation was found to be closely associated with obesity-related metabolic diseases. This association between obesity/type 2 diabetes and inflammation can be traced back to case reports published over a century ago, showing that high-dose sodium salicylate could diminish glycosuria in older diabetic patients (1, 2). Thereafter, several studies also showed that acetylsalicylic acid or sodium salicylate reduced the glucose level and improved glucose tolerance in diabetic patients (3, 4). These reports again drew attention in 1993 with the publication of a report demonstrating in mice that the expression of TNFα in adipose tissue was increased during the development of obesity, while conversely the neutralization of TNFα attenuated insulin resistance (5). Subsequently, the same research group demonstrated that TNFα suppressed insulin signaling by inhibiting insulin receptor tyrosine kinase activity (6) and proposed a model in which inflammation defined as an increased level of TNFα in adipose tissue could be the basis of systemic insulin resistance. Concurrently with these findings, leptin was identified as a secretory bioactive molecule from adipocytes, which regulates food intake and energy expenditure through the hypothalamus (7). This led to the establishment of an innovative concept in which adipose tissue not only simply stores excess energy as triacylglycerol but is also an organ that secretes the biologically active substances referred to as adipokines. Adipokines could directly regulate the insulin sensitivity of remote insulin-sensitive organs including liver and skeletal muscle through the circulation. Deregulated adipokine secretion from the expanded adipose tissue of obese individuals was shown to contribute to the development of systemic insulin resistance and metabolic diseases. Following the discovery of leptin (7), a number of adipokines have been identified; these include IL-6 (8, 9), resistin (10), retinol-binding protein 4 (RBP-4) (11), omentin (12), chemerin (13–15), progranulin (16), and monocyte chemoattractant protein-1 (MCP-1) (17–19). The proinflammatory cytokine TNFα, produced mainly by macrophages that have infiltrated into adipose tissue, can also be considered as an adipokine (5, 20). Given that TNFα activates proinflammatory signal cascades as well as inhibits insulin receptor signaling, this molecule is thought to be a major player linking adipose tissue inflammation and insulin resistance (21, 22). In contrast, in a lean state, a certain level of “healthy” adipokines contributes to insulin sensitivity and adequate glucose homeostasis. For instance, adiponectin is considered a “healthy” adipokine. Adiponectin-deficient mice exhibited insulin resistance (23, 24) along with increased expression of TNFα in adipose tissue (23). Chronic inflammation, especially in adipose tissue, causes impairment of adipokine secretion, leading to systemic insulin resistance. Thus, adipose tissue inflammation and adipokine secretion are strongly associated with each other and coordinately contribute to insulin resistance in obesity.

## Macrophage Accumulation in Adipose Tissue

The mechanisms by which TNFα is increased during obesity were unclear until the findings published in 2003 that chronic inflammation observed in rodents and humans was characterized by the accumulation of macrophages into adipose tissue (21, 22). In general, macrophages differentiate in tissue from recruited monocytes and function in innate immunity during host defense. However, these studies demonstrated that macrophages existed even in a lean state, but expanded their populations during the development of obesity in mice and humans (21, 22). It is now considered that macrophages defined as F4/80^+^ CD11b^+^ are resident in lean adipose tissue, representing 5% of the stromal vascular fraction (17, 25), but are increased by obesity up to 14–30% (17, 18, 25). In healthy subjects, adipose tissue macrophages show dynamic diversity. Kosteli et al. showed that, although chronic weight loss reduced the macrophage content in adipose tissue, fasting or acute weight loss in turn elicited their accumulation (26). Such conditions seemed to enhance the lipolysis that caused elevation of local free fatty acid (FFA), which induced macrophage accumulation. Infiltrated macrophages incorporate lipids, which act to suppress lipolysis. These findings provide evidence that, not only in a pathological state, but also in physiological circumstances, macrophages in adipose tissue play dynamic roles in the maintenance of homeostasis.

## The Role of Chemokines in Adipose Tissue Inflammation and Insulin Resistance

**Figure 1:**
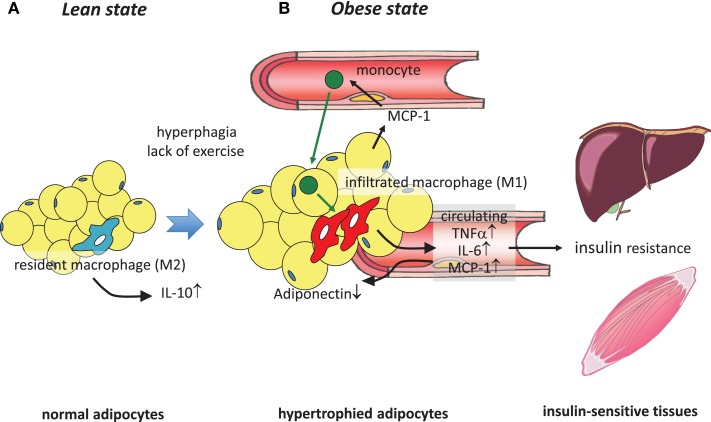
**Obesity-induced macrophage infiltration into adipose tissue causes insulin resistance**. **(A)** In adipose tissue in a lean state, most resident macrophages are M2 macrophages that contribute to insulin sensitivity by secreting IL-10. **(B)** Hyperphagia and lack of exercise cause hypertrophy of adipocytes, which induces MCP-1 secretion to the circulation, leading to the recruitment of circulating monocytes to adipose tissues. These infiltrated monocytes differentiate into activated M1 macrophages, which robustly secrete proinflammatory cytokines such as TNFα, IL-6, and MCP-1, thus contributing to low-grade inflammation in adipose tissue and a decrease of adiponectin. At the same time, these secreted cytokines cause insulin resistance in liver and skeletal muscle by acting as insulin resistance-inducing adipokines.

Chemokines are a family of low-molecular-weight proteins with an essential role in leukocyte trafficking during both homeostasis and inflammation. On the basis of their molecular structure, chemokines are divided into two major subgroups: CC chemokine ligand (CCL) and CXC chemokine ligand (CXCL), which bind to CC chemokine receptor (CCR) or CXC chemokine receptor (CXCR), respectively (27). Intriguingly, MCP-1 (also known as CCL2), a representative CC chemokine, was found to be remarkably increased in adipose tissue in obesity (21, 22, 28). We and others sought to investigate whether MCP-1 is a factor that enhances the infiltration of macrophages in adipose tissue. Adipose tissue-specific overexpression of MCP-1 in mice indeed increased macrophage infiltration into adipose tissue and insulin resistance (17, 19), whereas disruption of MCP-1 or its receptor, CCR2, impaired high-fat diet (HFD)-induced migration of macrophages into adipose tissue, thereby reducing adipose tissue inflammation and attenuating insulin resistance (17, 18, 29, 30). These findings suggest that MCP-1 secreted from enlarged adipocytes attracted circulating monocytes to adipose tissue, causing inflammatory characteristics of adipose tissue. Infiltrated monocytes differentiate into macrophages and produce additional inflammatory cytokines, leading to further inflammation. Secreted inflammatory cytokines are supposed to induce insulin resistance in liver and skeletal muscle by functioning as adipokines (Figure 1). In addition, chronic increase in the circulating level of MCP-1 by the administration of recombinant MCP-1 protein induced insulin resistance, macrophage infiltration into adipose tissue, and an increase in hepatic triacylglycerol content without affecting body weight (18). Acute increase in the circulating MCP-1 concentration also induced insulin resistance but not macrophage infiltration into adipose tissue. These findings indicate that an increase in the concentration of MCP-1 in the circulation is sufficient to induce systemic insulin resistance irrespective of adipose tissue inflammation (18). In fact, circulating MCP-1 levels were found to be increased in type 2 diabetic patients compared with normal subjects (31, 32) or to be correlated with HOMA-IR in type 2 diabetic patients (33). On the other hand, studies by others found no difference or even more infiltrated macrophages in adipose tissues in MCP-1-deficient mice, although the reason for the different results is unknown (34, 35). Recent study by Oh et al. provided evidence by employing a new method for quantitative *in vivo* macrophage tracking, in which monocytes isolated from peripheral blood were labeled *ex vivo* with fluorescent PKH26 dye and then injected into recipient mice (36). Mice receiving CCR2-deficient monocytes were protected from HFD-induced accumulation of macrophages in adipose tissue and the liver, while transplantation of intact monocytes into MCP-1 knockout mice on an HFD did not cause infiltration of macrophages into the tissues (36). These results all suggest that the MCP-1-CCR2 signaling pathway plays an important role in adipose tissue inflammation (17–19, 29, 30, 36), hepatic steatosis (17, 18, 37, 38), and glucose metabolism (17–19, 29, 30, 36–38) in insulin-resistant model mice. Thus, examination of the factors that induce MCP-1 expression in hypertrophied adipocytes is also important. Ito et al. demonstrated that down-regulation of mitogen-activated protein kinase (MAPK) phosphatase-1 (MKP-1) increased MCP-1 expression through MAPK activation in cultured adipocytes (39). Furthermore, Kitade et al. demonstrated that the expression of CCR5 in adipose tissue was similarly increased during obesity (40). Genetic deletion of CCR5 in mice resulted in protection against HFD-induced macrophage infiltration, insulin resistance, and hepatic steatosis. Furthermore, alteration of macrophages in adipose tissues was accompanied by polarization to M2. These results were reproduced using a cell-specific approach by employing bone marrow transplantation (40). At present, it is believed that M2 macrophages contribute to maintain insulin sensitivity, while obesity causes a switch to M1 polarization that enhances systemic insulin resistance through the secretion of inflammatory cytokines (41). Subsequently, the contributions of chemokines other than the CCL family, such as CXCL14 (42) or other factors including osteopontin (43), angiopoietin-like protein 2 (Angptl2) (44), serum amyloid A (45), and dietary cholesterol (46), to the accumulation of macrophages in adipose tissue have been demonstrated.

## Inflammatory Activation of Myeloid Cells in the Liver

Following the findings for adipose tissue, the issues of whether obesity can cause hepatic inflammation and whether this inflammation can contribute to hepatic or systemic inflammation became important in this field. Obesity-associated nutrient excess has been linked to inflammation in part via activation of inhibitor of κB kinase β (IKKβ) and subsequent nuclear translocation of nuclear factor κB (NF-κB), one of the key transcriptional mediators of inflammation (47–49). Consumption of an HFD clearly induced proinflammatory activation of Kupffer cells, the resident macrophages of the liver, in mice (50, 51). In addition, inflammatory activation of Kupffer cells was implicated in the pathogenesis of obesity-induced insulin resistance and fatty liver disease (50). Deletion of IKKβ in myeloid cells reduced macrophage-mediated inflammation and improved obesity-associated systemic and hepatic insulin sensitivity (47). Furthermore, chemical deletion of Kupffer cells was demonstrated to cause improved insulin sensitivity during HFD feeding (52). Obesity and insulin resistance are often associated with hepatic steatosis in a large proportion of obese patients. We demonstrated mechanically that overexpression of MCP-1 in adipose tissue caused hepatic steatosis along with adipose tissue inflammation, while systemic deletion of MCP-1 inhibited HFD-induced steatosis (17). In addition, chronic increase of plasma MCP-1 level was also sufficient to induce hepatic steatosis and adipose tissue inflammation (18). These findings suggest that an increase of circulating MCP-1 or adipose tissue inflammation may cause hepatic steatosis. Although HFD feeding caused M1 activation of Kupffer cells in the liver (50, 51), it seemed that the number of Kupffer cells was not increased in obesity (53). Using flow cytometry, it was investigated how a population of myeloid cells (CD11b^+^) changed during obesity or type 2 diabetes. Kupffer cells, defined as CD45^+^, F4/80^+^, were a major subset of myeloid cells in the liver. Obesity rather reduced the number of Kupffer cells, while in turn, the proportion of myeloid cells, defined as CD11b^+^, CD45^+^, F4/80^low^, doubled, from 10.0 to 19.7% (53). Given that these recruited myeloid cells were also characterized by CCR2^+^, hepatic expression of CCL2/CCR2, which was increased by HFD, seemed to have originated from infiltrated macrophages. By employing bone marrow transplantation from CCR2-deficient mice, it was further demonstrated that the trafficking of the infiltrated cells was dependent on CCR2. In addition, adenoviral overexpression of CCL2 in the liver caused the accumulation of myeloid cells coincident with hepatic steatosis (53). CCR2-dependent recruitment of myeloid cells to the liver (36) and CCL2-dependent development of hepatic steatosis (54) were also demonstrated by other studies. These results also underline the role of the CCL2-CCR2 signaling pathway in the recruitment of myeloid cells to the liver. Taking these findings together, the range of immune cells in the liver is thus complex and heterogeneous, but they are thought to play important roles in both insulin resistance and hepatic steatosis.

## Regulation of Kupffer Cell Activation by Endothelial NO Production

**Figure 2:**
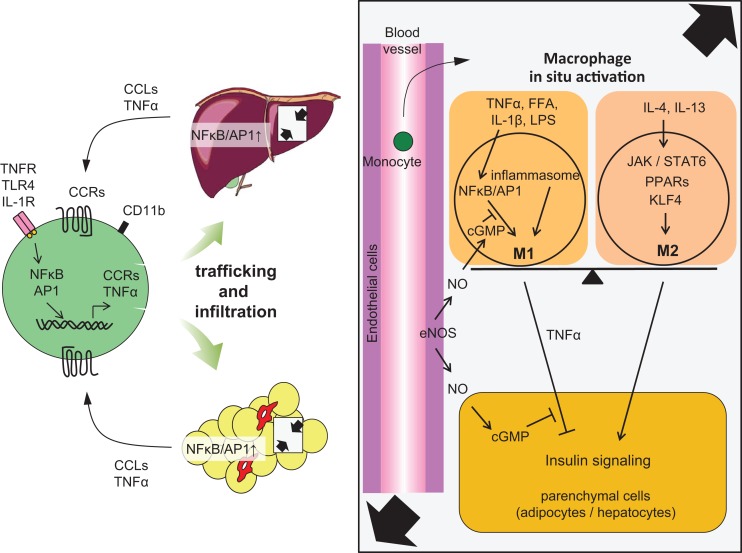
**Accumulation of monocytes/macrophages in adipose tissue and liver, and activation in the tissues**. Trafficking: During obesity, adipocytes exhibit hypertrophy, while liver incorporates substantial FFAs, both of which cause tissue inflammation, activation of NF-κB, and AP1 signaling, leading to increased secretion of inflammatory chemokines and cytokines, including CCLs and TNFα. Elevated secretion of CCLs (e.g., MCP-1) elicits the accumulation of CCR-positive monocytes to the site of inflammation, particularly CCR2^+^ for adipose and liver, but CCR5^+^ for adipose tissue. *In situ* activation: In a lean state, resident tissue macrophages display the M2 phenotype, which is achieved and sustained through the JAK/STAT6 pathway in response to IL-4 or IL-13 stimuli. These stimuli are derived from resident T_H_2 cells, T_reg_ cells, eosinophils, and mast cells. PPARs and KLF4 also induce M2 activation. In turn, obesity and subsequent elevation of tissue FFA or inflammatory cytokines stimulate NF-κB and AP1 signaling, which causes switching of the phenotype to M1, leading to further secretion of TNFα. Signal from inflammasome also activates M1 activation. M1 activation of macrophages can be suppressed by endothelial NO/cGMP signaling. M2 macrophages contribute to insulin sensitivity in neighboring parenchymal cells, while M1 induces insulin resistance, with the M1/M2 balance determining tissue and/or systemic insulin sensitivity.

Local and systemic insulin resistance has been discussed in relation to the interactions between immune cells and parenchymal cells. We have proposed that endothelial cells could be added to those components with which interactions are shown. We have demonstrated that HFD feeding induced proinflammatory activation of Kupffer cells in wild-type (WT) mice coincident with reduced liver endothelial nitric oxide synthase activity and nitric oxide (NO) content while, conversely, the enhancement of cGMP signaling downstream of endogenous NO by phosphodiesterase-5 inhibition protected Kupffer cells against HFD-induced inflammation (51). Furthermore, proinflammatory activation of Kupffer cells was evident in eNos^−/−^ mice, even on a low-fat diet. Targeted deletion of vasodilator-stimulated phosphoprotein (VASP), a key downstream target of endothelially derived NO, similarly led to a predisposition to hepatic and Kupffer cell inflammation and abrogated the protective effect of NO signaling in both macrophages and hepatocytes studied in a cell culture model (51). These results collectively imply a physiological role for endothelial NO to limit obesity-associated inflammation and insulin resistance in hepatocytes and support a model in which Kupffer cell activation during HFD feeding is dependent on reduced NO signaling (51) (Figure 2). The NO/cGMP/VASP axis was also shown to be relevant in adipose tissue (55).

## Constituent Cells Other than Macrophages in Obesity-Induced Inflammation: Interactions Among Immune Cells During Inflammation in Adipose Tissue

**Figure 3:**
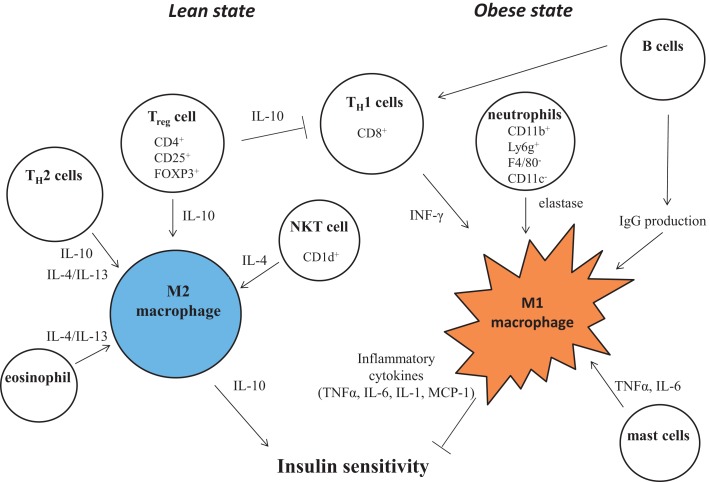
**Control of M1/M2 polarization by neighboring immune cells**. In a lean state, resident T cells consist of T_H_2 cells, T_reg_ cells, and NKT cells. Combined with resident eosinophils, these cells sustain the M2 activation of macrophages through secreting IL-4, IL-10, and IL-13. As obesity progresses, alteration of constituent immune cells occurs, in which the numbers of T_H_2 cells and T_reg_ cells decline, while in turn, T_H_1 cells and B cells increase. In addition to these more prevalent cells, neutrophils and mast cells induce M1 activation of macrophages by increased secretion of elastase, TNF, IFNγ, IL-6, and pathogenic IgG. B cells also activate T cells.

The role of macrophages in adipose tissue inflammation has been clearly demonstrated. Besides these cells, additional leukocyte subpopulations have recently been demonstrated to be involved in obesity and insulin resistance, such as T cells, B cells, eosinophils, neutrophils, mast cells, and natural killer cells. The involvement of multiple leukocyte subpopulations underlines the complexity of obesity-associated adipose tissue inflammation (Figure 3).

### T cells

Although macrophage infiltration in adipose tissue has been demonstrated in both mice and humans (56), little is known about the sequence of events that lead to the macrophage accumulation in adipose tissue. Research attempting to investigate which surface antigens of immune cells are associated with inflammation and insulin resistance revealed the involvement of CD11c-positive cells (57). Myeloid-specific deletion of CD11c in mice protected against HFD-induced accumulation of macrophages in adipose tissue and exhibited insulin sensitivity compared with the controls (57). Next, T cells (CD4^+^, CD8^+^) were found to be increased in adipose tissue during obesity (58–60). In a lean state, CD4^+^ helper T cells and regulatory T (T_reg_) cells (CD4^+^, CD25^+^, Foxp3^+^) were predominant; however, prior to the accumulation of macrophages (F4/80^+^, CD11b^−^), CD8^+^ T cells infiltrated coincidentally with a reduction of the number of T_reg_ (25). The administration of CD8 antibody to WT mice fed an HFD attenuated macrophage infiltration and insulin resistance. Although CD8 knockout mice were protected against HFD-induced accumulation of macrophages, restoration of CD8^+^ T-cells increased macrophage infiltration. Similar results were obtained by other groups (61, 62). It is now considered that, in a lean state, CD4^+^ CD25^+^ Foxp3^+^ T_reg_ cells induce alternative activation of monocyte/macrophages (63), which is characterized by the expression of macrophage mannose receptor (MMR) or intracellular activity of arginase (64). T helper type 2 (T_H_2) cells expressing IL-4 and IL-13 also induce M2 activation of macrophages that secrete IL-10, whereas macrophages are M1-activated through IFNγ by T helper type 1 (T_H_1) cells and through IL-17 by T_H_17 cells. Recently, peroxisome proliferator-activated receptor γ (PPARγ) activity in T_reg_ cells has been shown to be important to reduce chronic inflammation in adipose tissue (65).

### B cells

The accumulation of B cells was observed in adipose tissue of mice fed an HFD before macrophage and T-cell accumulation (66). In addition, diet-induced obese mice lacking B cells were protected from metabolic abnormalities despite weight gain (67). B-cell effects on glucose metabolism were associated with the activation of proinflammatory macrophages and T cells and the production of pathogenic IgG antibodies. In fact, treatment of mice fed an HFD with a B-cell-depleting CD20 antibody ameliorated abnormality in glucose metabolism and adipose tissue inflammation, whereas the transfer of IgG from mice with diet-induced obesity rapidly induced insulin resistance and glucose intolerance (67). Recently, obese B-cell-null mice were reported to exhibit decreased systemic inflammation, inflammatory B- and T-cell cytokines, adipose tissue inflammation, and insulin resistance compared with obese WT mice (68). This was associated with an increased percentage of anti-inflammatory regulatory T cells. B cells from type 2 diabetes subjects promote proinflammatory T-cell function through contact-dependent mechanisms, suggesting that B cells regulate inflammation in type 2 diabetes by modulating T-cell functions (68).

### Eosinophils

In addition to T_H_2 or T_reg_ cells, eosinophils have been shown to exist in lean adipose tissue and participate in the maintenance of M2 activation through secreting IL-4 (69). By using eosinophil-deficient and hypereosinophilic mice, Wu et al. showed that eosinophil-derived IL-4 and IL-13 determined the M2 activation of macrophages in adipose tissue and contributed to insulin sensitivity. Hypereosinophilic mice displayed improved insulin sensitivity, while eosinophil-deficient mice exhibited increased fat together with impaired glucose tolerance and insulin resistance (69).

### Neutrophils

Neutrophils are rare in a lean state; however, an HFD elicits the accumulation of neutrophils (CD11b^+^ Ly6g^+^ F4/80^−^ CD11c^−^), which seem to induce local insulin resistance by secreting elastase (70). The deletion of neutrophil elastase in HFD-induced obese mice led to reduced macrophage content and inflammation. These changes were coincident with improvement of glucose tolerance and increased insulin sensitivity. Intriguingly, neutrophil elastase can degrade IRS-1 protein and cause insulin resistance in adipocytes (70). Similar results were obtained in a very recent study by another group (71). In humans, an increased blood level of myeloperoxidase, a marker of neutrophils, in obese women (72), and increased activity of neutrophils in obese subjects have also been noted (71, 73).

### Mast cells

Mast cell invasion was also detected in adipose tissue in obese mice (74). Mast cell-deficient mice (*KitW-sh/W-sh* mice) were protected from HFD-induced body weight gain and the increase of proinflammatory cytokines and chemokines along with the improvement of glucose metabolism and energy expenditure due to the up-regulation of UCP-1 expression in BAT (74). Similar effects were observed in the treatment of mice with a mast cell-stabilizing agent. Mast cells were supposed to promote diet-induced obesity and glucose intolerance by the production of IL-6 and IFNγ. Mast cells are also involved in obesity-induced adipose tissue inflammation and insulin resistance. Weight gain of mast-cell-deficient mice during HFD was decreased compared with that of control mice (75). Mechanistically, prostaglandin J2 (PGJ2) produced by mast cells in response to high-glucose enhanced adipocyte differentiation by PPARγ activation, leading to obesity (75).

### Natural killer T cells

Natural killer T (NKT) cells are innate-like T lymphocytes that recognize glycolipid antigens and have been implicated in autoimmunity, microbial infection, and cancer and hence represent an important immunotherapeutic target (76). Similar to eosinophils, NKT cells have been shown to reside in lean adipose tissue, in which they contribute to sustain the M2 activation of macrophages by stimulating IL-4/STAT6 signaling (77, 78). Schipper et al. demonstrated that CD1d-null mice whose NKT cells were not activated displayed a distinctive insulin resistance phenotype even on a low-fat diet without overt adipose tissue inflammation (79). Activation of NKT cells has thus been demonstrated to modulate polarization toward M2, resulting in improved glucose metabolism (78–80). Unlike in mouse studies, the role of NKT cells during obesity and adipose tissue inflammation in humans remains unclear. An unaltered number of circulating NKT cells in obesity (80, 81) and significantly lower numbers of circulating NKT cells in obese patients have been documented (82).

## Cell Signaling in Macrophages that Defines M1 and M2 Activation

Macrophages are terminally differentiated cells of the mononuclear phagocyte system that include dendritic cells, circulating blood monocytes, and committed myeloid progenitor cells in the bone marrow. Local environmental factors are known to affect the properties, functions, and activation state of macrophages. In general, macrophage activation is defined across two separate polarization states, M1 (proinflammatory) and M2 (anti-inflammatory) states. M1 or “classically activated” macrophages are induced by proinflammatory mediators such as lipopolysaccharide (LPS), TNFα, and IFN-γ. M1 macrophages are also associated with enhanced proinflammatory cytokine production (TNFα, IL-6, IL-1). On the other hand, M2 or “alternatively activated” macrophages have low proinflammatory characteristics and instead generate high levels of anti-inflammatory cytokines, for example, IL-10. Since the attenuation of macrophage M1 activation and the maintenance of M2 activity are believed to be important for intact glucose metabolism, there has been research focusing on intracellular signaling that determines proinflammatory or alternative activation in macrophages (Figures 2 and 3).

### M1 macrophages

M1 activation of macrophages is established mainly through the IKKβ/NF-κB and Jun N-terminal kinase (JNK) 1/activator protein 1(AP1) system. Obesity induces adipose tissue inflammation, which results in high levels of proinflammatory cytokines and chemokines. In particular, TNFα is a representative inflammatory cytokine that causes lipolysis in adipose tissue. Thereby, plasma FFA levels are usually elevated in obesity. FFAs released from adipocytes through lipolysis have been shown to be capable of serving as ligands for the toll-like receptor 4 (TLR4) complex (83). TLRs are initially indispensable for innate immune cells to recognize intruding pathogens and trigger an appropriate immune response. Among them, TLR4 is a high-affinity receptor for LPS, which is a component of the cell walls of gram-negative bacteria (84). TLR4 signaling activated by FFA induces the expression of a large number of proinflammatory target genes and drives M1 activation by regulating the transcriptional factors including NF-κB, AP1, and interferon-regulatory factor (IRF) family members. TNFα also drives M1 activation by inducing proinflammatory genes through activating NF-κB and AP1 transcriptional factors. For instance, lipid infusion caused the accumulation of macrophages in adipose tissue accompanied by insulin resistance in WT control mice, but this was not the case in TLR4-deficient mice (83). Hematopoietic cell-specific deletion of TLR4 in mice attenuated HFD-induced insulin resistance in adipose and the liver (85). Activated TLR4 signaling induced a classical inflammatory response, which led to the recruitment of macrophages. In this way, macrophages activated to M1 by FFA through TLR4-mediated signaling secrete TNFα, which in turn enhances lipolysis in neighboring adipocytes, leading to further production of FFA. This vicious cycle or paracrine loop mediated by TNFα and FFA between adipocytes and macrophages in obese adipose tissue induces further adipose tissue inflammation (86). In addition, TNFα and FFA inhibit insulin receptor signaling via the increase of serine phosphorylation of IRS-1. Recently, a liver secretory glycoprotein, fetuin-A, was demonstrated to play a crucial role as an endogenous ligand for TLR4 in FFA-induced inflammation and insulin resistance in adipocytes (87). The serum concentration of fetuin-A was significantly increased in obese diabetic patients compared with that in non-obese non-diabetic human subjects. Next, myeloid differentiation primary response protein 88 (MYD88), the primary mediator of TLR and IL1 receptor signaling, has been investigated to clarify whether this is also involved in the FFA-induced insulin resistance. However, MyD88 deficiency in mice exacerbated diet-induced glucose intolerance and hyperlipidemia (88). There is therefore a conflict regarding the activity of the TLR4/MyD88 axis in diet-induced obesity and insulin resistance, which remains to be elucidated in future studies.

### M2 macrophages

The activation of M2 macrophages is basically maintained by the signaling of the IL-4/JAK/STAT6 pathway. The administration of IL-4 to mice induces M2 activation of macrophages, thereby attenuating HFD-induced insulin resistance (89). IL-10 secreted by M2 macrophages enhances insulin signaling, including that in the liver, thereby having a protective role against obesity-induced insulin resistance (90). Taking these findings together, the activation of IL-4 signaling is considered to be a promising target to suppress insulin resistance and thus studies to identify molecular mediators are underway. We describe here several factors involved in M2 activation.

#### Peroxisome proliferator-activated receptor γ

Macrophage-specific deletion of PPARγ in mice impaired M2 activation despite the mice being on a chow diet (91). In these mice, adiponectin expression was decreased. This change was accompanied by reduced oxidative phosphorylation in liver and skeletal muscle, which might have contributed to the insulin resistance in these tissues. Another study demonstrated that macrophage-specific PPARγ-deficient mice showed glucose intolerance and insulin resistance in a lean state. These mice had increased inflammatory markers in adipose tissue, liver, and skeletal muscle and showed decreased effects of thiazolidinediones, indicating a requirement for PPARγ in macrophages for intact insulin sensitivity in muscle/liver and a full antidiabetic effect of thiazolidinediones (92). Odegaard et al. demonstrated that peroxisome proliferator-activated receptor δ (PPARδ) mediated the effects of a Th2 cytokine, IL-4, to direct the expression of the alternative phenotype in Kupffer cells and adipose tissue macrophages of lean mice (50). Adoptive transfer of PPARδ^−/−^ bone marrow into WT mice conversely diminished the alternative activation of hepatic macrophages, causing hepatic dysfunction and systemic insulin resistance (50). Collectively, PPARs are thought to be required for the maturation of M2 activation and the resulting insulin sensitivity.

#### Krüppel-like factor 4

In addition to PPARs, another nuclear receptor, Krüppel-like factor 4 (KLF4), has been implicated in M2 activation in macrophages (93). In macrophages, KLF4 is suppressed by LPS stimulation, while it is increased by IL-4. Macrophage-specific KLF4 knockout mice display M1 activation and M2 disactivation. Owing to reduced fatty acid oxidation, the mice are susceptible to becoming obese and exhibit glucose intolerance and insulin resistance. In contrast, forced expression of KLF4 in RAW cultured macrophages resulted in M2 activation and resistance to M1 polarization by stimulation of LPS. Importantly, mRNA expression of KLF4 in adipose tissue is reduced in human obesity. Moreover, mRNA expression of KLF4 is not only positively associated with adiponectin expression in adipose tissue but also with well-defined M2 markers, such as CD206 and CCL18 in the stromal vascular fraction of adipose tissue. It has also been documented that IL-4 activates STAT6, leading to transcriptional activation of KLF4 to induce M2 genes (93).

#### AMP-activated protein kinase

AMP-activated protein kinase (AMPK) is an evolutionarily conserved sensor of cellular energy status that is activated by low energy status (increased cellular AMP/ADP:ATP ratio) and consists of an α catalytic subunit and βγ regulatory subunits. This molecule has also been shown to be crucial for the maintenance of M2 activation (94). Galic et al. tested the effect of AMPK β1 loss in macrophages *in vivo* by transplantation of bone marrow from WT or β1(−/−) mice into WT recipients. When challenged with an HFD, mice that received β1(−/−) bone marrow displayed enhanced adipose tissue macrophage inflammation and liver insulin resistance compared with animals that received WT bone marrow (94). Taking these findings together, the activation of AMPK and increased fatty acid oxidation in macrophages might provide an avenue for the treatment of type 2 diabetes.

#### Sirtuin 1

Sirtuin 1 (SIRT1), the mammalian homolog of yeast silent information-regulator 2 (Sir2), is an NAD^+^-dependent histone deacetylase that has been implicated in the regulation of lifespan under calorie restriction (95) or energy metabolism during fasting (96); thus, it is believed to be a promising target for type 2 diabetes (95, 97). Besides these findings, anti-inflammatory effects have also been demonstrated, showing that SIRT1 deacetylates NFκB and suppresses its transcriptional activity by inhibiting nuclear translocation (98). SIRT1 levels are markedly reduced in adipose tissue of obese humans and mice (99, 100). HFD was also found to result in cleavage of SIRT1 protein (101). In fact, upon the reduction of SIRT1 in fat by antisense oligonucleotides to levels similar to those seen during overnutrition, macrophage recruitment to adipose tissue was significantly increased. Similar results were obtained in fat-specific SIRT1 knockout mice. In contrast, overexpression of SIRT1 in mice prevented HFD-induced accumulation of macrophages (102). Furthermore, it was found that the SIRT1 expression level in human subcutaneous fat was inversely related to the number of adipose tissue macrophages. Mechanistically, others demonstrated that SIRT1 regulated intracellular inflammatory signaling at the levels of JNK and IKK (103). In addition, AMPK was also reported to regulate lipid-induced inflammation negatively through SIRT1 (104). Taken together, these findings indicate that SIRT1 might exert an insulin-sensitizing effect partially through the suppression of inflammation.

## Involvement of Inflammasome in Obesity-Induced Inflammation

The mechanisms by which obesity induces macrophage activation despite the absence of any infection or autoimmune processes remained unclear. Although some mechanisms including hypoxia (105, 106) and autophagy (107–109) have been proposed for the induction of inflammation, in this review, we would like to focus on a new concept, the involvement of inflammasome in adipose tissue inflammation and insulin resistance. External or internal stimuli are recognized by pattern recognition receptors (PRRs). External stimuli, particularly pathogen-associated molecular patterns (PAMPs), are detected not only by TLRs but also by inflammasome, which is a protein complex consisting of caspase-1, apoptosis-associated speck-like protein containing a caspase recruitment domain (ASC), and nucleotide-binding oligomerization (NOD)-like receptors (NLRs) (110). Among these components, different pathogens are recognized by distinct constituents of NLRs. For instance, bacterial infection is recognized by nucleotide-binding domain, leucine-rich-containing family, pyrin domain-containing-1 (NLRP1), NLRP3, NLRP4, and absent in melanoma 2 (AIM2). Viral infection is recognized by NLRP3 and AIM3. Fungal or parasitic infection is recognized by NLRP3 (110). But all of these infections cause the activation of caspase-1, which eventually leads to the processing and secretion of proinflammatory cytokines, including IL-1β and IL-18 (110). A unique feature of inflammasome is its additive ability to recognize internal stimuli as danger signals. For instance, uric acid, silica, fatty acids, and ATP in cytoplasm are detected as non-microbial-originated damage-associated molecular pattern molecules (DAMPs) by NLRs (110). Since mRNA expression of NLRP3 in adipose tissue correlates with IL-1β, body weight, and blood glucose level in rodents and humans (111), Vandanmagsar et al. tested whether NLRP3 played important roles during the development of chronic inflammation in obesity. Using NLRP3 knockout mice, they showed that NLRP3 sensed ceramide as a danger signal that activated caspase-1, which enhanced IL-1β secretion, thereby inducing T-cell activation (111). Target deletion of NLRP3 in mice displayed improved glucose tolerance and increased insulin sensitivity. These results were accompanied by the appearance of small adipocytes, reduced M1 activation, and enhanced insulin signaling in liver, adipose tissue, and skeletal muscle. Elevated ceramide, saturated fatty acid, reactive oxygen species (ROS), and mitochondrial dysfunction caused activation of inflammasome in macrophages (108, 112). The resulting activation of caspase-1 and subsequent secretion of IL-1β then interfere with insulin signaling, whereas inhibition of caspase-1 has been demonstrated to attenuate insulin resistance coincident with improved function of adipocytes (108, 112, 113). In humans, elevated levels of circulating IL-18 in patients with type 2 diabetes have been demonstrated (114), along with a suppressive effect of calorie restriction and resulting weight loss on the reduced expression of adipose NLRP3 in type 2 diabetes (111), and marked reduction of both adipose and liver expression of IL-1β in morbidly obese subjects by laparoscopic adjustable gastric banding surgery (114).

## Therapeutic Interventions

The basis of therapeutic interventions in inflammation and insulin resistance is to prevent or to ameliorate obesity by physical exercise and diet control. They can also present the beneficial effects to the improvement of inflammation irrespective of body weight loss. In addition, the significance of chronic inflammation and its molecular mechanisms during the development of type 2 diabetes has been demonstrated and, in mice, suppression of inflammation-related molecules has successfully improved glucose intolerance. On the basis of this evidence, clinical trials targeting inflammation-related molecules have started. Thus, at first we would like to introduce the contribution of exercise and diet to the amelioration of inflammation. Next, we describe the current circumstances concerning several clinical applications of anti-inflammatory drugs.

### Exercise and diet

Although exercise is generally admitted to be effective to attenuate obesity and sustain health, single session of exercise has been reported to trigger an increase in proinflammatory cytokine release together with leukocytosis and increased plasma concentration of CRP (115). Regular and chronic exercise, however, has been reported to be associated with reduction of inflammatory markers such as CRP, IL-6, and TNFα (115–118). Physical (aerobic + resistance) exercise was also associated with increase in anti-inflammatory substances, such as IL-4 and IL-10 in type 2 diabetic patients with metabolic syndrome (118). Among many types of exercise, Oliveira et al. compared the effect of 12 weeks training with three different types of exercise (aerobic training, strength training, and combined training) on subjects with type 2 diabetes, demonstrating that the aerobic training program caused significant up-regulation in antioxidant enzymes (119). Accordingly, exercise-dependent improvement of glucose tolerance seems to be related with suppression of inflammation and oxidative stress (116).

Dietary calorie restriction is well recognized to be beneficial to ameliorate obesity-induced inflammation through weight loss. In addition to this, dietary composition has also been demonstrated to be important for the improvement of inflammation. Dietary bioactive compounds, such as polyphenols and certain fatty acids suppress systemic and adipose tissue inflammation. Polyphenols such as resveratrol exhibited anti-inflammatory effects via suppression of NF-κB (120) and extracellular signaling regulated kinase pathway (121) as well as via activating SIRT1 (122). Resveratrol has also been shown to activate AMPK independent of SIRT1 (123). Therefore, resveratrol may be a promising candidate in anti-inflammatory therapy (124). For instance, resveratrol supplementation for 30 days decreased blood glucose levels and inflammation markers along with improvement of HOMA index in healthy obese men irrespective of body weight (125). In addition, dietary polyunsaturated fatty acids, such as eicosapentaenoic acid (EPA) or docosahexaenoic acid (DHA) possess anti-inflammatory effects. Mechanistically, these include activation of AMPK and PPARγ (126). These polyunsaturated fatty acids also inhibits NF-κB pathway by activation of G-protein coupled receptor (GPR) 120 (127). In fact, n-3 polyunsaturated fatty acids (EPA and DHA) decreased adipose tissue and systemic inflammation in severe obese non-diabetic patients and improved lipid metabolism (128). EPA was demonstrated to reduce body weight at least by suppressing lipogenesis in the liver (129).

### Clinical applications of anti-inflammatory drugs

#### Aspirin/salsalate

It has been reported that high-dose sodium salicylate or acetylsalicylic acid could diminish glycosuria or improve the blood glucose level in diabetic patients (1–4). Given that IKKβ is a key downstream mediator of insulin resistance and its blockade by salicylates attenuated hyperglycemia, hyperinsulinemia, and dyslipidemia in obese rodents (130, 131), Hundal et al. asked whether high-dose aspirin (∼7 g/day) could ameliorate insulin resistance and improve glucose tolerance in patients with type 2 diabetes (132). They demonstrated that this treatment for 2 weeks resulted in marked reduction of metabolic parameters including fasting glucose, basal rate of hepatic glucose production, and insulin-stimulated peripheral glucose uptake, despite no change in body weight. A large randomized trial, the National Institute of Diabetes and Digestive and Kidney Diseases-sponsored Targeting Inflammation with Salsalate, Non-acetylated Prodrug of Salicylate, in Type 2 Diabetes (TINSAL-T2D) trial, recently concluded that salsalate lowered hemoglobin A1c (HbA1c) levels and improved glycemic control in patients with type 2 diabetes (133). In a single-masked run-in period, patients were randomly assigned to receive placebo or salsalate at a dosage of 3.0, 3.5, or 4.0 g/day for 14 weeks (27 patients each) in addition to their current therapy. Mean HbA1c changes were −0.36% (*P* = 0.02) at 3.0 g/day, −0.34% (*P* = 0.02) at 3.5 g/day, and −0.49% (*P* = 0.001) at 4.0 g/day compared with placebo (133). The number of patients studied and the trial duration were insufficient to warrant recommending the use of salsalate for type 2 diabetes; however, it appears warranted to target this molecule in further investigations.

#### IL-1β

Reducing the activity of inflammasome and suppressing IL-1β secretion might be targets to attenuate insulin resistance in diabetes. Randomized clinical trials have shown that the blockade of IL-1β signaling by anakinra, a recombinant human IL-1 receptor antagonist, reduced systemic inflammation and improved glycemia of type 2 diabetes (134–136).

#### TNFα

Etanercept is a dimeric recombinant form of the extracellular domain of the human p75 TNFα receptor 2 fused to the Fc fragment of human immunoglobulin G1 (IgG1) and acts as a TNFα antagonist by interfering with the binding of TNFα to its cellular receptors and thus blocks the inflammatory response (137). Several studies have been conducted to test whether this biopharmaceutical improves glucose tolerance in patients with type 2 diabetes; however, despite a suppressive effect on systemic inflammation, the attenuation of glucose tolerance or insulin resistance has not yet been achieved (137–139). These results might be attributable to the distinct role of TNFα between rodents (5) and humans (137–139). Alternatively, antagonism of TNFα by other drugs remains hopeful in future studies.

## Concluding Remarks

Following the discovery of chronic inflammation characterized by macrophage accumulation in adipose tissue, an explosion of studies in the past decade have begun to reveal the contributions of inflammation to the development of insulin resistance and subsequent metabolic abnormalities in other tissues, such as liver (47–52) and most recently brain (140). Adipose tissue, liver, and the hematopoietic system are evolutionarily derived from the same tissue. This developmental heritage can underlie the link between obesity-induced adipose tissue and hepatic inflammation (56) (Figure 2). Studies using flow cytometry subsequently identified the relative importance of other immune cells, including T cells, B cells, eosinophils, neutrophils, mast cells, and NKT cells, during the development of chronic inflammation. At present, besides the identification of constituent immune cells, an avenue intended to reveal how these neighboring immune cells modulate the inflammatory signals in macrophages has being created. In order to reveal the significance of inflammation during the development of type 2 diabetes, the identification of both factors that regulate trafficking of macrophages and intracellular molecules that control inflammatory activation in macrophages would be indispensable. Since there might be substantial differences in the nature of inflammation between rodents and humans and since clinical applications have not yet achieved excellent results, the question remains of how much the inhibition of inflammation contributes to improving glucose homeostasis. In future, there is a need for translational research that applies evidence from mice to human subjects. Because chronic inflammation is also involved in the development of atherosclerosis, rheumatoid arthritis, cancers, and neurodegenerative diseases, the suppression of inflammation can be a desirable therapy for type 2 diabetes. However, simple reduction of inflammation cannot be a beneficial approach as innate immunity is a radical form of homeostasis to deal with pathogenic infections. In addition, since the pathophysiology does not develop via a single molecule, multilayered targeting of various molecules without affecting physiological immune function has to be achieved. The location, timing, and degree of suppression all have to be controlled. Although recent studies have shed light on the pathophysiological roles of inflammation in diabetes, substantial efforts are required to achieve clinical application in human subjects.

## Conflict of Interest Statement

The authors declare that the research was conducted in the absence of any commercial or financial relationships that could be construed as a potential conflict of interest.
